# News and Notes

**Published:** 1905-01

**Authors:** 


### *NEWS AND NOTES.*

---

THE German Supreme Court forbids the use of the title "Doctor" in Germany, when granted in the United States.

---

DR. ANITA MCGEE and her party of American trained nurses have been decorated by the Mikado for their service in the hospitals in Japan.

---

AN International Congress on Tuberculosis will be held in Paris October 2 to 7, 1905. The president of the pathological section will be Dr. Lannelongue, and the social section, Dr. Landouzy.

---

SMALL BOY—"Mister, ma wants ter know if you'll please stop your auto in front of our house fer half an hour. She thinks th' smell from your autermobeel may drive away th' mosquitoes."—*Judge.*

---

THE one hundred and fiftieth anniversary of the founding of King's College was celebrated by the trustees of Columbia University on Friday, Saturday, Sunday, and Monday, October 28 to 31.

---

"COFFEE HEART" is a term lately added by medical examiners for life-insurance companies to their regular classification of the functional derangements of that organ. Its effect is in the shortening of the long beat of the heart.

---

DR. THOMAS DARLINGTON, Health Commissioner of New York, has selected for the study of diseases of the lungs Drs. Edward G. Janeway and T. Mitchell Pruden of New York, Drs. W. H.

Welch and Wm. Osler of Baltimore, and Dr. Theobald Smith of Boston.

---

THE plush seat covering ordinarily used by the railway and street-car systems of the country, has been placed under State ban in Kentucky. The Board of Health will indict every railroad management that does not henceforth employ leather or cane seat covering in coaches.

---

THE LIKELY COMBINATION.—Young Rooney: "Do yez t'ink two kin live as chapely as wan?"

Old Cassidy: "Phwat's th' idea?"

Old Cassidy: "And phwat's 'two' got t' do wid it, ye fule? Ye shud figger on eight or tin, me bye!"—*Puck.*

---

THE Pennsylvania Railroad has just adopted a plan for providing first aid to the injured. It consists of equipping all baggage, mail, express, construction, and wrecking cars, terminals, yard offices, shops, and important stations with stretchers; and locomotives, yard offices, shops, and important stations with "first-aid boxes."

---

A LITTLE GIRL was heard talking to her rabbit. "Five times five," she said. "Six times six, seven times seven. Between times she shook the rabbit violently. "Dorothy," said her mother, "what are you doing to your rabbit?" "Well, papa says," replied the child, "that rabbits multiply rapidly and Bunny won't do it."—*Town Talk.*

---

CAPT. SALVATORE PIZZATI, a philanthropic citizen of New Orleans, has announced his intention to give $250,000 toward the erection of a hospital "for the alleviation of suffering which shall stand as a monument to the Italian colony in New Orleans and which shall be maintained for the honor and glory of God." The plans for the hospital are being drawn, and it is estimated that the institute will cost $1,000,000.

A REFORMED German spoke at a temperance meeting as follows: "I shall tell you how it vas. I put my hand on my head; dere vas von big pain. Den I put my hand on my pody and dere vas anoder. Den I put my hand in my pocket and dere vas nodings. Now dere is no more pain in de head. De pain in my pody vas all goned avay. I put my hand in my pocket, and dere is twenty dollars. So I shtay mit de temperance."—*Ex.*

---

THE superior jury of awards of the Louisiana Purchase Exposition has awarded to Dr. S. A. Knopf, of New York City, a gold medal for his work on tuberculosis. The exhibit consisted of Dr. Knopf's English, French, and German text-books on tuberculosis, his contribution on tuberculosis in the Twentieth Century Practice of Medicine, his various addresses and lectures, and his international prize essay, "Tuberculosis as a Disease of the Masses and How to Combat It," with translations in eighteen languages.

---

THE Fourth Pan-American Medical Congress, which will convene in Panama the first week in January, next, bids fair to be a most delightful mid-winter trip. The delegates will leave this country by the Atlantic, Pacific and Gulf Coasts the last week in December. They will return by the same routes, or will make round trips.

The public Health Association meeting will take place on the following week in Havana, and those desirous of attending both meetings, can arrange to do so.

There are two routes for the physicians to take from Panama to Havana. The first is by way of Jamaica to Santiago de Cuba by boat and overland by rail to Havana. The second is by water from Panama to Vera Cruz and from there to Havana. The former will probably be the most pleasant trip.

From Havana, the return trip can be made directly north to New York by water or via Miami or Tampa, Florida, or New Orleans. The connections and dates of sailing are now being arranged.

The Panamanian Government has appropriated the sum of $25,-000 for the Scientific Session and the entertainment. The Congress will be held from the second to the sixth of January. The afternoons will be devoted to the Scientific Sessions and the mornings and evenings to trips and social functions. So far as can be learned, the program in Panama will be a reception on the first day, by President Amador of the Panama Republic, and the formal opening of the session of the Congress the same evening. On the second day, an excursion to the Canal in the morning, meeting of the various sections in the afternoon, and a banquet in the evening; on the third day, an excursion down the bay to Taboga Island, where a Panama breakfast will be served, scientific sessions in the afternoon, and a ball in the evening. On the fourth day, an excursion to the U. S. Army barracks in the morning, section meetings in the afternoon, and the formal closing session in the evening. On the fifth day, an excursion to the plantation of the United States Fruit Company; and on the afternoon of this day, those of the congresistas who intend going to Cuba to attend the meeting of the Public Health Association will sail for Jamaica, while those who intend going by way of Vera Cruz, or returning home by way of New Orleans or New York, will remain until the following Tuesday.

The Secretaries of the Sections of the Congress for the United States, are: Drs. A. H. Doty, of New York, hygiene and quarantine; Judson Daland, of Philadelphia, medicine; R. Matas, of New Orleans, general surgery; Bert Ellis, of Los Angeles, eye; Hudson Makuen, of Philadelphia, throat; Frederick Jack, of Boston, ear; C. H. Hughes, of St. Louis, nervous diseases; Geo. Goodfellow, of San Francisco, military surgery; John Ridlin, of Chicago, orthopedic surgery; D. W. Montgomery, of San Francisco, dermatology; C. G. Kerley, of New York, pediatrics; Noble P. Barnes, of Washington, therapeutics; Walter Chase, of Boston, pathology.

Communications from physicians in the United States, interested in these branches, can be sent directly to these different secretaries. Delegates intending to attend the Congress, desirous of obtaining information concerning it, should communicate with the Secretary of the International Executive Committee in the United States, Dr. Ramon Guiteras, 75 West 55th St., New York City.

